# Report of Surgical Cases at the Buffalo General Hospital

**Published:** 1862-09

**Authors:** J. R. Lothrop

**Affiliations:** Attending Physician


BUFFALO

### Medical and Surgical Journal

VOL. II. SEPTEMBER, 1862. NO. 2.

### ORIGINAL COMMUNICATIONS.

ART. I.—*Report of Surgical Cases at the Buffalo General Hospital. By* J. R. LOTHROP, *Attending Physician.*

10. The fractures under treatment were, of the humerus two, of the clavicle one, of the fibula two. The fractures of the humerus were, one of the external condyle, and one of the shaft two inches above the condyles, ununited.

The first occurred in a man sixty years of age, of intemperate habits. While intoxicated he fell heavily, striking upon the elbow, the elbow itself bearing marks which left no doubt of its being caused by a direct blow. The fracture penetrated deeply into the joint, probably opening into it near the middle of the articulating surface. Movement of the radius by rotation or by pulling it forwards caused crepitus, as did pressing the condyles together. The breadth between the condyles was increased and the fragment was pushed slightly backwards. A bandage had been applied before the patient was seen by me, which had been so much tightened by the swelling as to cause slight vesications. The arm was therefore laid on a pillow, in a flexed position, and cold lotions applied for the first few days, until the swelling, which was considerable, had somewhat subsided and the vesications healed. It was then dressed on a paste-board splint, flexed at a right angle. The splint was placed upon the anterior aspect of the arm, not from any preference for that surface—many, perhaps most surgeons, choosing the posterior surface—but because it was free from vesications. The dressings were removed often, and passive motion employed. The splint was continued longer than is generally advised—three or four weeks—but as the paste-board had lost its firmness, it allowed quite free motion. The result of this fracture was bony union, slightly delayed, with a partial restoration of the motions of the joint. At the present time, five months after the accident, flexion and extension, though somewhat limited, are more perfectly established than rotation and supination, which are imperfect. In this fracture the danger is less from displacement, than stiffness of the joint, or even bony anchylosis, which may follow if passive motion is neglected after the first seven or eight days.

The second was a case of ununited fracture of two years' duration. The fracture was caused by a gun-shot wound. Two months before he came under my care, Dr. Miner had performed an operation for the purpose of procuring union. I was present and assisted at the operation, therefore describe it as I saw it, and also the primary dressing. An incision on the external aspect of the arm, down to the ends of the bone, was made; the ends turned outwards, and removed by the saw. In this way, about three-fourths of an inch of each was removed. The cut surfaces of the bone were then placed in apposition, and tied by a silver wire. After the closure of the wound, the arm was confined, flexed, to a firm pasteboard splint. The inflammation which followed was considerable, attended with free suppuration. As the patient was a healthy young man, a better result than followed seemed highly probable. The ends of bone removed, were rounded and covered by a smooth fibrous covering. The case remained under the care of Dr. Miner till the beginning of my term of service. At that time, two months after operation bony union had not taken place, though there was no mobility of the fragments on each other. The arm could be bent at the point of fracture, yet it was firm enough to indicate a considerable amount of plastic material about the ends. At first there was considerable discharge of pus, not only at the incision, but at new openings which had been established. This, however, gradually diminished, so that at the end of a month it had ceased entirely. The arm was dressed on an angular splint, though had it not been in that position previously, a straight splint would have been preferred. But as the swelling which followed the operation had somewhat stiffened the joint, it was thought an attempt to extend the arm would separate the fragments and diminish the probability of union, not very great at best. The patient left the hospital after about nine or ten weeks, in sound health, but not with bony union, though the hope of its occurring had not been abandoned. In this connection, we may notice, as deserving attention, the opinion which has been advanced, that in fractures of the humerus at its lower third, dressing upon a straight splint affords the most favorable conditions for the prevention, as well as for the treatment of the non-union which so frequently occurs in this fracture.

The fracture of the clavicle took place at the outer end of the middle third, its direction downwards and inwards, the inner fragment overlapping the outer, and forming a prominence even after union. It was caused by a counter stroke on the shoulder. The dressing combined the axillary pad and the sling, two things spoken of as very important in the treatment of this fracture. The humerus was kept, as nearly as possible, in line with the body, the elbow being carried more backwards than is directed in the use of Fox's dressing. It would be more proper to say this was attempted, for experience must have convinced most surgeons of the difficulty, if not impossibility, of keeping any retentive apparatus in its place. The result in this case was a shortening of nearly one-half an inch; a result as good as is usually obtained by any retentive dressing, and upon the whole not much better than is realized when the very simplest methods are employed, in fractures at this point. It has not been very clearly shown that any dressing will prevent overlapping, and a little shortening does not interfere with free use of the arm, though it may cause slight deformity. The patient left the hospital in about twenty days, with good union, and soon after resumed labor.

Of the two fractures of the fibula, one was recent, and one of several weeks' duration. The first—occurring in a young man—was caused by a mis-step, in getting out of a street car, the foot, as the patient declared, turning *inwards*. The point of fracture was about three inches above the ankle joint, was followed by considerable swelling about the joint; but there was no turning out of the foot, or perceptible deformity. It therefore required only to be kept at rest for a short time, two or three weeks, during which time motion was occasionally made of the ankle joint, to prevent the stiffness which usually follows. In five or six weeks the patient began to walk about, though the joint continued somewhat stiff and troublesome for a time.

The second, occurring in a man forty-five years of age, was likewise caused by a mis-step in getting out of a vehicle, the ground being covered with ice at the time. In this case the foot turned *outwards* at the time of the accident, and was followed by great deformity, the foot inclining outwards, and a marked prominence of the inner malleolus existing. The joint, moreover, was quite stiff. The point of fracture was about two and one-half inches above the joint. The fracture had occurred several weeks before the admission of the patient. With little hope of remedying the deformity, strong extension by means of a twisted cord was made, with motion of the joint to break up the stiffness. Afterwards it was dressed on Dupuytren's splint, on the inside of the leg, with a thick pad just above the internal malleolus, and a few turns of a roller around the foot below the external malleolus, to rotate the foot strongly inwards. This dressing was continued several weeks; at the end of which time, the deformity was somewhat diminished. It should, however, be stated, that this observation was made while the patient was still in bed. The difficulty in preventing, by careful management, the turning out of the foot, should lead us to distrust apparent improvement, in the attempt to remedy it. Though the foot may remain quite straight while the patient is in bed; getting up, and bearing the weight upon it, may in a short time restore the original deformity. As the patient left the hospital at that time no opportunity has been afforded to ascertain whether the improvement was permanent. He left, however, with a firm belief that he had been benefited.

11. The five cases of ulcers were varicose ulcers of the leg—four of them upon the left leg—as far as they go confirming the observation that the greater number of these ulcers is found upon the left leg, giving therefore some show of probability to the opinion, that the pressure of the distended sigmoid flexure of the colon, in persons habitually constipated, by obstructing the return of venous blood, acts the part of a cause. The ulcers varied in size from one to four inches in diameter. The treatment of these often obstinate cases was reduced to great simplicity; water dressing, if the patient would keep in bed, and adhesive straps and a bandage, if he insisted upon walking about. These two methods seem to be more useful to most cases, than all the variety of medicated applications, except at the very outset.

12. The instance of benign tumor, was a fibrous tumor of the face. The patient, a young man about twenty years of age, first observed it four years before, it being as large as a bullet. At the time of his admission it was about the size of an English walnut, deeply imbedded in the cheek, being much more distinct to the touch on the inside. It appeared to have an attachment under the prominence of the malar bone. The patient desired its removal, on account of the deformity it caused. An operation was therefore made for its removal. The incision was made in an oblique direction across the cheek in a line from the angle of the mouth to the prominence of the malar bone, high enough to avoid the salivary duct.

On account of its depth and the smallness of the incision, the dissection was difficult, especially for the removal of the pedicle. It was followed by a troublesome hæmorrhage, which was finally controlled by the pressure of a sponge, as in the narrow and deep wound a ligature could not be applied. The tumor was firm, inelastic, of almost cartilaginous consistence. When divided it was of a uniform whitish color. The tumor belonged to the class of fibrous tumors, histologically, though in physical properties it had much resemblance to cartilage. This was evident from its slow growth, and entire freedom from pain during its progress, as well as from its structure.

13. The diseases of the bones and joints were, one of anchylosis, one of caries and one of traumatic inflammation of the knee joint; one of chronic synovitis and one of necrosis.

Of the first, which occurred in a youth as the result of scrofulous inflammation, involving the bones, it is only necessary to say, that there was no attempt made to remedy it, the boy receiving constitutional treatment.

The case of caries of the knee joint was one in which an opening had been made into the joint for the evacuation of pus. An account of the case, and the results to the date of this report, may be found in a former number of this journal. The report concludes with the statement that the knee was greatly benefited by the operation; that the boy was able to move about; and seemed in all respects improving rapidly. Shortly before he came under my care, while playing about the ward, he received an injury to the knee from a fall, which obliged him to keep in bed, and to dispense with the hinged splint, worn while he was up. At the beginning of my term the knee was much swollen, and discharged pus very copiously. This with the pain which accompanied, had very much impaired the boy's strength, and given rise to great irritative fever. During the three months succeeding, there were periods of apparent improvement, but the latter part of the time, pus was discharged through several openings. Fever, pain, and loss of appetite had so much exhausted him, that his parents thinking he would soon die, removed him from the hospital. At the present time, however, he is still living; but there is extensive disorganization of the joint, partial dislocation, and continued suppuration; neither have the other symptoms improved. The treatment was mainly directed to the quieting of irritation and pain, and sustaining strength. For these purposes, opium, iron, and wine were made use of. An attempt was made to keep the limb at rest by a paste-board splint, but the amount of discharge prevented its continued use. The course of this case cannot be looked upon as unusual, but is spoken of more at length, because the opening into the joint seemed to promise permanent improvement. In speaking of the result, due allowance must be made for the injurious influence of the violence inflicted by the fall. There can be no doubt of the propriety of an incision for the escape of pus when confined in the cavity of a joint. The error lies rather in not making it sufficiently free. If permanent improvement does not follow, and the result is unfavorable, nothing of it is to be charged upon the operation; and on the other hand, changes for the better do not warrant a very confident belief that they will continue.

The case of inflammation of the knee resulted from a cut by an axe, about two inches above the patellæ. The wound was about two inches in length and deep, as was stated. At first it had progressed so favorably that the patient—a man forty years of age—removed from Grand Island to Buffalo. In consequence, inflammatory symptoms set in. When the patient entered the hospital, two weeks after their occurrence, the whole limb was enormously swollen, and discharging pus at the original wound. When the limb was kept at rest, semiflexed, and supported by pillows there was little pain, but motion caused most intense suffering. The constitutional symptoms were severe, great irritative fever, profuse sweatings, worn and anxious look, and altogether an aspect most unfavorable. Large poultices were applied to the limb, opium given to quiet irritability, and stimulants with good diet, to sustain strength. In a few days there were evidences of a large collection of pus in the calf. This being evacuated by incision, the limb began slowly to improve. The amount of pus which followed the incision was nearly a pint, and the quantity discharged during each day afterwards was very great. It collected in the upper and posterior portion of the calf, instead of working its way down among the muscles. At the present time, three months after admission, the discharge of pus has ceased, and the limb is reduced very nearly to the same size as the other. The leg, however, cannot be extended, though there is a very limited motion. In this case it is probable that the inflammatory action did not involve the bones, and though the joint was implicated to some extent, the parts around the joint must have been affected mainly, and the suppuration, confined to them. There is, therefore, every probability of a restoration of motion by forcible extension, if not by the use of the limb.

The case of chronic affection of the synovial membrane of the knee was of more than a year's standing. It arose from exposure to cold. In the outset there was swelling and effusion, with pain and interference with the motion of the joint; still, the symptoms were never very acute. The swelling had continued in its present form, from the beginning, liable to slight variations. Occasionally upon unusual exertion, or exposure to cold, pain was experienced, and especially while walking, which was at such times difficult both from pain and a degree of stiffness. Ordinarily, however motion was quite free and painless. The joint was considerably swollen, the swelling being most marked in front of the lower end of the femur, extending up under the extensor muscles, though it existed also on each side of the ligamentum patellæ. The swelling was soft, but did not impart a distinct sense of fluctuation to the touch. It was probably due mainly to thickening of the synovial membrane. It was at first treated by an application of the tincture of iodine, which, failing of effect, repeated blisters were employed. The latter removed entirely the pain which was felt at the beginning of treatment, and considerably reduced the swelling. After a little time the patient was able to move about freely, without inconvenience, and in two months left the hospital.

The case of necrosis involved the lower end of the femur, with incarceration of a sequestrum, which was removed by operation. The patient was a young man of nineteen years of age, who had been for some time under the care of Dr. Storck. Six years before, he met with an injury just below the knee, by striking against hot iron, so that a burn was added to the injury, Swelling and inflammation followed, which extended above the knee, and continued for about a year, during which time he suffered great pain. About a year after the injury, a discharge of pus took place above the knee, on the outer side of the thigh. This discharge has continued ever since, being at first considerable in amount, but soon becoming small in quantity. At the time of admission there was but a slight discharge. He had suffered scarcely any pain after the first year, and had lately worked at his trade, of painting. Upon examination the femur was found to be increased in diameter at its lower end, but its articulating extremity was unaffected, as the mobility of the joint was perfect. An opening had existed on the inner side of the thigh, but had not been permanent, opening and closing. An opening existed on the outside of the thigh towards the posterior aspect, a little way in front of the tendons of the ham, about three inches above the joint. Through this a probe passed in a direction inwards and forwards, to a depth of three inches, passing through a rough canal, and bringing up on rough and moveable bone. There could be no doubt that a sequestrum existed, but as the patient was in good health, had perfect use of the joint, suffered no pain either at rest or in walking, and was not greatly troubled by the amount of pus which escaped, there certainly was no *necessity* for an operation. But the patient desired to be rid of the little trouble he experienced. After consultation with my colleagues, Drs. Eastman and Miner, an operation was deemed advisable, the liability of some bad result not being sufficient to deter from it. The operation was made April 23d, the patient being under the influence of sulphuric ether. A longitudinal incision was made on the outside of the thigh, and a short transverse one backwards into the old opening. After preparing the way by dissection, the trephine was applied and a small circle of bone removed. Through the opening thus made several pieces of necrossed bone were removed. The circle of bone removed was about one-half an inch in thickness. The wound was closed by a few stitches and water dressing applied. For a few days after the operation there was considerable fever. The thigh was moderately swollen in the vicinity of the wound, and red lines extended up the thigh to the groin, where several glands were swollen and painful. All threatening symptoms disappeared in the first three or four days. Considering the severity of the operation the patient suffered but little, and in a short time was free from pain, had a healthy wound with slight discharge, and recovered his usual appetite. May 6th, following, he was dressed and about the ward. June 1st, the escape of pus had nearly ceased, and June 25th he left the hospital to resume his former employment.

It may occur to many to ask, whether in a case attended with such slight inconvenience, for there was surely nothing more; no pain, no impediment to motion or labor, no unfavorable influence upon the health, we may say no liability of the patient's being called to endure either; moreover, a case in which, in time Nature would have effected a cure; it was proper to advise an operation which might have been followed by severe inflammation, not only of the surrounding textures, but of the bone itself, carrying with it not only the liability to further death of bone, but even absorption of pus. Probably it cannot be claimed in this any more than in many other cases of greater magnitude, that the fortunate result shows the wisdom of the decision; but it may perhaps be safely stated that in operations of this nature, on bone and structures, which have been a long time subject to a morbid process, the danger of bad results from fresh inflammation is not very great. A bone which has become accustomed to tolerate a foreign body, as a piece of dead bone in effect is, is not likely to resent more actively the process for its removal.

